# Variability of Virgin Olive Oil Phenolic Compounds in a Segregating Progeny from a Single Cross in *Olea europaea* L. and Sensory and Nutritional Quality Implications

**DOI:** 10.1371/journal.pone.0092898

**Published:** 2014-03-20

**Authors:** Ana G. Pérez, Lorenzo León, Mar Pascual, Carmen Romero-Segura, Araceli Sánchez-Ortiz, Raúl de la Rosa, Carlos Sanz

**Affiliations:** 1 Department of Biochemistry and Molecular Biology of Plant Products, Instituto de la Grasa, CSIC, Seville, Spain; 2 IFAPA, Centro Alameda del Obispo, Cordoba, Spain; SERGAS, Santiago University Clinical Hospital, IDIS Research Laboratory 9, NEIRID Lab, Spain

## Abstract

Virgin olive oil phenolic compounds are responsible for its nutritional and sensory quality. The synthesis of phenolic compounds occurs when enzymes and substrates meet as olive fruit is crushed during the industrial process to obtain the oil. The genetic variability of the major phenolic compounds of virgin olive oil was studied in a progeny of the cross of Picual x Arbequina olive cultivars (*Olea europaea* L.). They belong to four different groups: compounds that included tyrosol or hydroxytyrosol in their molecules, lignans, flavonoids, and phenolic acids. Data of phenolics in the oils showed that the progeny displayed a large degree of variability, widely transgressing the genitor levels. This high variability can be of interest on breeding programs. Thus, multivariate analysis allowed to identify genotypes within the progeny particularly interesting in terms of phenolic composition and deduced organoleptic and nutritional quality. The present study has demonstrated that it is possible to obtain enough degree of variability with a single cross of olive cultivars for compounds related to the nutritional and organoleptic properties of virgin olive oil.

## Introduction

The beneficial effects of the traditional Mediterranean diet on human health have been widely reported. This diet reduces the risk of a number of diseases, mainly those containing an inflammatory component such as cardiovascular disease, certain types of cancer, diabetes, metabolic syndrome, arthritis and Alzheimer’s disease [1]–[2]. Olive oil is one of the oldest known plant oils and it is unique among them since it can be consumed as a fruit juice called virgin olive oil (VOO). This product represents the primary dietary lipid source in the Mediterranean diet and it has been also linked with its positive health benefits. Recent attention has been given to the phenolic fraction of VOO [1], [3], [4]. The long term dietary consumption of VOO would deliver the phenolic compounds over time which may attenuate the inflammatory response the human body undergoes when eating, and reduce the associated risk of chronic inflammatory disease states [1]. However, phenolics are important not only from a nutritional point of view but also in terms of sensory quality. Thus, the increase in the demand for high-quality VOO in the last years can be attributed not only to its potential health benefits but also to its unique organoleptic properties. VOO phenolics are responsible for the bitter and pungent sensory notes of this oil [5]–[7]. Bitterness and pungency are common and desirable attributes in VOOs when present at low to moderate intensity, but they are rejected by consumers when present at high intensity. Due to their health promoting and organoleptic properties, phenolic compounds are currently being used as quality markers for VOO and also as a trait in new cross breeding programs [8]. Therefore, the aim of increasing the quality standards for VOO is continuously stimulating the study of the biochemical pathways related to the nutritional and organoleptic properties and the search for new olive cultivars with an improved quality.

The synthesis of phenolic compounds responsible for the nutritional and sensory quality of VOO occurs when enzymes and substrates meet as olive fruit is crushed during the industrial process to obtain the olive oil. There are at least thirty-six structurally distinct phenolic compounds so far identified in VOO. Among them, hydrophilic phenols such as phenolic alcohols, phenolic acids, lignans, flavonoids and secoiridoids are the most important class of natural antioxidants found in both olive fruits and VOOs. There are many variations in phenolic profiles among VOOs [9], [10] as a result of an array of factors that depend on the intrinsic characteristics of the olive fruits, the edafo-climatic conditions, and the technological conditions used during olive oil processing. Although the latter may influence the phenolic profile of VOO [11], the composition and biochemical status of the olive fruit are the most important variables determining the synthesis of the VOO phenolic compounds during the oil extraction process. In this sense the presence of phenolic compounds in VOO is directly related to the content of phenolic glycosides initially present in the olive fruit tissues and the activity of hydrolytic and oxidative enzymes acting on these glycosides [12], [13]. The main phenolic glycosides found in the olive fruit are oleuropein, ligstroside and demethyloleuropein, although many others such as verbascoside, an elenolic acid glucoside, luteolin-7-glucoside, apigenin-7-glucoside and rutin have also been identified in fruits from different cultivars and maturation stages [14], [15]. The secoiridoid derivatives resulting from the enzymatic hydrolysis of oleuropein, ligstroside and demethyloleuropein, identified as the dialdehydic forms of decarboxymethyloleuropein and decarboxymethylligstroside aglycones (3,4-DHPEA-EDA and *p*-HPEA-EDA, respectively) and the aldehydic forms of oleuropein and ligstroside aglycones (3,4-DHPEA-EA and *p*-HPEA-EA, respectively) are the most abundant phenolic components found in most olive oils [16], and among them those derived from oleuropein display the strongest antioxidant activity [17]. These secoiridoid derivatives contain in their molecules the phenolic alcohol tyrosol (*p*-HPEA) or its hydroxyl derivative hydroxytyrosol (3,4-DHPEA). Partial hydrolysis of VOO main phenolic compounds during gastric and intestinal digestion has been widely described [18], [19], which increases especially 3,4-DHPEA concentration at the colonic level. Thus, extensive investigation has focused on 3,4-DHPEA as a chronic disease preventive agent.

The purpose of the present study was to make a screening of the major VOO phenolic compounds and deduced organoleptic and nutritional properties in a segregating population of the cross of Picual x Arbequina olive cultivars. This was carried out in the frame of an olive breeding program with the aim of identifying new olive cultivars which give rise to oils with an improved sensory and nutritional quality.

## Materials and Methods

### Plant material

A total of 136 olive (*Olea europaea*) seedlings from the cross Picual x Arbequina were considered in the present study. The two parents, Picual and Arbequina, were grown in the same orchard than the seedling progeny. Cross was made in spring 2001 and the obtained seedlings were submitted to the habitual protocol followed in the breeding program [20]. Initial seedling growth was forced in greenhouse by means of drip fertirrigation, temperature control and continuous light. Plants were established in open field in September 2003 at 1,5×4 m spacing, trained to form the canopy at 160 cm height, and then developed freely. Drip irrigation and standard cultural practices were followed to ensure tree growth without limitations. Trees were grown in the same edafo-climatic conditions at the experimental orchards of IFAPA Alameda del Obispo, Córdoba, Spain. Fruits were picked by hand when reaching an average ripening index of 2,5 (turning stage) for better comparison of genotypes according to El Riachy *et al*. [21], during three consecutive years (2008-2010).

### Olive oil extraction

Olive oil was extracted using an Abencor analyzer (Comercial Abengoa, S.A., Seville, Spain) that simulates the industrial process of VOO production at lab scale [22]. Milling of olive fruits was performed using a stainless steel hammer mill operating at 3000 rpm provided with a 5 mm sieve. Malaxation was carried out for 30 min with the Abencor thermo-beater operated at 30 °C according to industry recommendations. Centrifugation of the kneaded paste was performed in a basket centrifuge at 3500 rpm for 1 min. After centrifugation, the oils were decanted and paper filtered. Oils were stored under nitrogen at -20°C until analysis.

### Extraction and analysis of virgin olive oil phenolic compounds

VOO phenolics were isolated by SPE on a diol-bonded phase cartridge (Supelco, Bellefonte, PA) following a previously described procedure [23]. A solution of *p*-hydroxyphenyl-acetic acid (4.64×10^−2^ mg/mL) and *o*-coumaric acid (9.6×10^−3^ mg/mL) in methanol was used as internal standard in this extraction procedure. An aliquot (0.5 mL) of standard solution was added to each oil sample (2.5 g) before phenolic extraction. Two phenolic extracts were obtained from each VOO.

VOO phenolic extracts were further analyzed by HPLC in a Beckman Coulter liquid chromatographic system equipped with a System Gold 168 detector, a solvent module 126 and a Mediterranean Sea 18 column (4.0 mm i.d.×250 mm, particle size 5 μm) (Teknokroma, Barcelona, Spain) following a previously described methodology [24]. The quantification of phenols (except ferulic acid) and lignans was carried out at 280 nm using *p*-hydroxyphenyl-acetic acid as internal standard. The quantification of flavones and ferulic acid was done at 335 nm using *o*-coumaric acid as internal standard. The identification of compounds was confirmed by HPLC-MS using the same chromatographic system connected on-line with a MAT95 magnetic sector mass spectrometer (Finnigan Mat, Bremen, Germany) equipped with an ESI-II electrospray inonization (ESI) interface with the same column and gradient conditions. The ESI mass spectra in the positive mode were obtained under the following conditions: capillary temperature, 220°C; lens, skimmer, and octapole voltages were set to get optimal response for a pattern solution of reserpine. Nitrogen at 200 kPa was used as the sheath gas. Afterward, partial defocusing of interface was done in order to generate moderate collision-induced dissociation (CID) inside the ionic transport region. Under these conditions, the spectra show enough ionic fragmentation to verify structural information from the protonated molecular ion.

### Statistical analysis

Data were statistically evaluated using STATISTICA (Statsoft Inc., Tulsa, OK, USA). Correlations among phenols or group of phenols were analyzed using Pearson’s correlations. Principal component analysis (PCA) was used to evaluate the levels of association among the phenol contents from the cross progeny. Factor analysis was performed using the normalized Varimax method.

## Results and Discussion

To date, olive breeding programs have been mainly focused on the improvement of agronomic traits, although more recently the major breeding targets have shifted more towards the sensory and nutritional qualities of VOO [8], [25]. Very recent studies that have focused on the sensory and nutritional parameters of olive oil have given more information and have considered further the concepts relating to their origins in the plant and their importance for human health [1], [2]. Taking into account the proven relationship between the phenolic composition of VOO and its benefits for human health, the major aim of the present study was to assess the phenolic composition of the oils from a segregating progeny of the Picual x Arbequina cross for over three consecutive years. Data was deposited at the Olegen web page (https://chirimoyo.ac.uma.es/oleagen).

**Figure 1:**
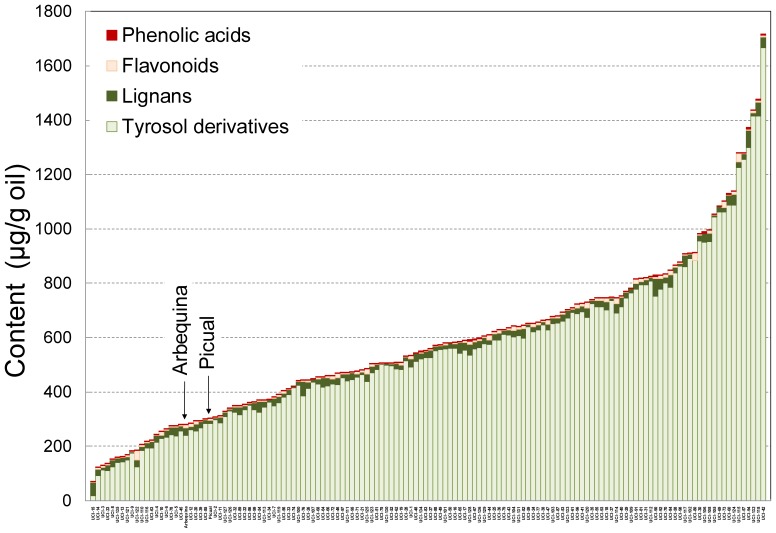
Main phenolic compound groups in the oils. Content of the main groups of phenolic compounds in the oils from the Picual x Arbequina progeny.

**Figure 2:**
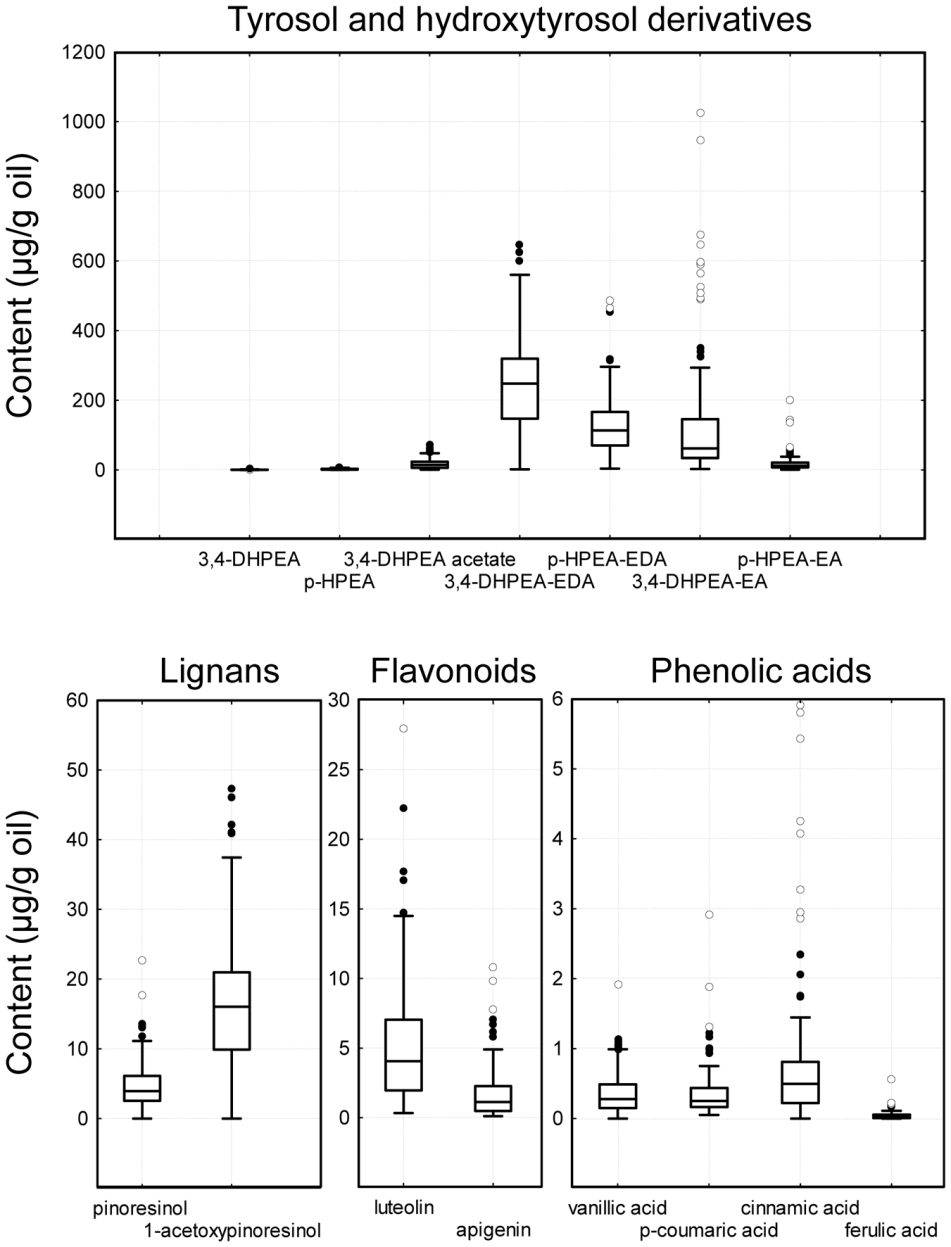
Ranges and distributions of main phenolic compounds in the oils. Ranges and distributions of the contents (μg/g oil) of tyrosol and hydroxytyrosol derivatives, lignans, flavonoids, and phenolic acids in the oils from the Picual x Arbequina progeny. Horizontal lines in the interior of the boxes are median values. The height in a box is equal to the interquartile distance, indicating the distribution for 50% of the data. The outliers (solid dots) and extreme data (open dots) are indicated outside the whiskers (the lines extending from the top and bottom of the box).

As shown in Figure 1, the progeny displayed a high degree of variability among individuals for the content of phenolic compounds, widely transgressing the genitor levels. Previous reports on segregation of the content of phenolic compounds on olive oil had shown only small amount of individuals with higher values than the parents [25], [26]. Actually, data from this progeny showed to have on average a higher content of phenolic compounds than the mentioned works and other breeding selections [8], [27]. The main phenolic compounds found in the progeny oils belong mostly to four different groups, compounds derived from *p*-HPEA and 3,4-DHPEA (tyrosol and hydroxytyrosol derivatives), lignans, flavonoids, and phenolic acids. As shown in Figure 1, most phenolics in the oils were tyrosol and hydroxytyrosol derivatives, whose contents were on average 25–400 times higher than those of the rest of phenolic groups in the oils. The most abundant compounds within the tyrosol and hydroxytyrosol derivatives were those with a secoiridoid chemical structure (3,4-DHPEA-EDA, *p*-HPEA-EDA, 3,4-DHPEA-EA and *p*-HPEA-EA) (Figure 2). Among them, 3,4-DHPEA-EDA was the most abundant on average. The mean content of this compound in the oils was 247 μg/g oil with a range of variability from 2 to 649 μg/g oil. Thus, 3,4-DHPEA-EDA turns out to be the main antioxidant in the oils due to its high level in the oils and its orthodiphenolic structure. Both *p*-HPEA-EDA and 3,4-DHPEA-EA showed the same average content in the progeny oils (134 μg/g oil) and they were visibly the most abundant phenolic in the oils after 3,4-DHPEA-EDA. However, whereas *p*-HPEA-EDA displayed a median value of 114 μg/g oil and a content range of 4-487 μg/g oil, 3,4-DHPEA-EA showed lower median value (62 μg/g oil) and higher range of variability (3–1024 μg/g oil). A lower content was observed for the other phenolic compound in the oils with a secoiridoid structure, *p*-HPEA-EA, with 19 μg/g oil mean value and a range of variability from 1 to 200 μg/g oil.

As mentioned before, *p*-HPEA-EDA and 3,4-DHPEA-EA have important nutritional and organoleptic properties [3]. *p*-HPEA-EDA, also known as oleocanthal, possesses similar anti-inflammatory properties to ibuprofen so that it is considered as one of the main factors within the Mediterranean diet reducing the risk of a number of diseases containing an inflammatory component [1]. More recently, Scotece et al. [28] also demonstrated that *p*-HPEA-EDA inhibits multiple myeloma cells proliferation. Additionally, *p*-HPEA-EDA seems to be the main phenolic responsible for the VOO pungency, producing a strong burning pungent sensation at the back of the throat, which is very important for VOO acceptation by consumers [7]. On the other hand, 3,4-DHPEA-EA seems to be the main compound responsible for the bitterness of VOO, which is also very important from the consumer point of view. Taking into account the equation for VOO bitterness calculation [bitterness  = 0.51+7.99 · (mmol 3,4-DHPEA-EA/kg oil)]found by Mateos et al. [6], at least 18% of the Picual x Arbequina progeny would give rise to oils with a maximum punctuation for bitterness (5 points). The rest showed to have a high variability for bitterness level. Thus, 48% of the progeny oils would not reach the score considered mild bitter (2 points) and 34% of the progeny oils would fall into the categories from mild to highly bitter. The bitterness calculations for the genitors Arbequina and Picual oils displayed values of 0.7 and 3.7, respectively, in good agreement with the experimental data found for these cultivars [6].

Among the tyrosol and hydroxytyrosol derivatives not displaying a secoiridoid structure, 3,4-DHPEA acetate showed the highest mean content in the progeny oils (18 μg/g oil) (Figure 2). This progeny also showed to give rise to oils with a high content variability of 3,4-DHPEA acetate with a value range of 1.3–71.5 μg/g oil. 3,4-DHPEA acetate has been reported to protect against oxidative DNA damage [29], oxidative stress in human cervical cells [30] and human hepatoma cells [31], and to possess anticancer activity against human adenocarcinoma [32]. This compound seems to be better absorbed in differentiated Caco-2 cell monolayers than its free counterpart 3,4-DHPEA [32] but, as far as we know, there are no data of its presence in plasma after sustained and moderate doses of VOO consumption as it has been demonstrated for 3,4-DHPEA and *p*-HPEA [33]. Although most studies relate the beneficial effect of VOO consumption with the level of 3,4-DHPEA in plasma [3] different beneficial effects of *p*-HPEA have been also widely demonstrated despite the lack of an orthodiphenolic structure and the consequent lower *in vitro* antioxidant activity compared to 3,4-DHPEA [34], [35], [36]. Contents of 3,4-DHPEA and *p*-HPEA in the progeny oils were the lowest among the tyrosol and hydroxytyrosol derivative group of phenolic compounds. The mean contents for 3,4-DHPEA and *p*-HPEA were 1.05 and 2.72 μg/g oil and the content value ranges of 0.15–5.65 μg/g oil and 0.45–9.88 μg/g oil, respectively.

Lignans represented on average the second major group of phenolics in the oils of the Picual x Arbequina cross progeny although they are at a concentration 25 times lower than those of the tyrosol and hydroxytyrosol derivatives (Figure 2). These are one of the major classes of chemical compounds referred to collectively as phytoestrogens, structurally similar to estradiol, which is the primary estrogen hormone in humans. Recently published research indicates that olive oil lignans, among other olive oil chemicals, may play an active role in protecting against breast cancer [37], [38]. As displayed in Figure 2, the most abundant lignan on average quantified in the progeny oils was1-acetoxypinoresinol. The mean value in the oils was 17.1 μg/g oil and the contents ranged in the interval 0-47.4 μg/g oil. Thus, there are individuals exceeding the levels of this lignan in the genitor Arbequina, which is characterized by a high level of 1-acetoxypinoresinol (36.4 μg/g oil). The mean value found for pinoresinol was 4.9 μg/g oil, close to the levels measured for the genitors Arbequina and Picual (4.8 and 6.2 μg/g oil, respectively). The content range for pinoresinol was 0–22.6 μg/g oil. These values are within the value ranges for different olive cultivars that can be found in the Phenol-Explorer database [39].

Taking into account the content in the oils, the third group of importance in the oils was the flavonoids. Flavonoids are important for human health because of their high pharmacological activities as radical scavengers and high antioxidant capacity in both *in vivo* and *in vitro* systems [40], [41]. Two main compounds were quantified, luteolin and apigenin. Luteolin was on average the major flavonoid quantified in the progeny oils (Figure 2), displaying a mean value of 5.13 μg/g oil and the contents ranged in the interval 0.35–27.90 μg/g oil. The mean value found for apigenin was 1.71 μg/g oil and the content range was 0.12–10.83 μg/g oil.

Finally, the last group of compounds measured was that of the phenolic acids. As shown in Figure 2, they were present in a very low concentration in the progeny oils. The mean content for the four phenolic acids quantified was 1.51 μg/g oil and the content range was 0.22–6.44 μg/g oil. Cinnamic acid was on average the main phenolic acid found in the oils. The *in vitro* antioxidant activity of phenolic acids depends on the number of hydroxyl groups in the molecule that would be strengthened by steric hindrance. The electron-withdrawing properties of the carboxylate group in benzoic acids (vanillic acid) have a negative influence on the H-donating abilities of the hydroxy benzoates. On the other hand, hydroxylated cinnamates (cinnamic, *p*-coumaric and ferulic acids) seem to be more effective for electron-withdrawing than the benzoate counterparts [42].

The relationships among the four groups of phenols in the Picual x Arbequina cross progeny oils (tyrosol and hydroxytyrosol derivatives, lignans, flavonoids, and phenolic acids) are summarized in Table 1. Overall, significant positive correlations were found among all the four groups of compounds for the oils. As expected, all four groups were significantly correlated with the total content of phenols. Tyrosol and hydroxytyrosol derivatives have the highest correlation coefficient (*r* = 0.999), while the other three groups of phenolics had modest correlation coefficients (*r* = 0.192–0.421). Moreover, in general the four groups of phenolics significantly correlated with the content of phenols either having or not an orthodiphenolic structure in the molecules (orthodiphenols and non-orthodiphenols, respectively).

**Table 1:** Pearson’s correlation coefficients among the main phenolic compounds found in the progeny Picual x Arbequina oils.

	3,4-DHPEA	*p*-HPEA	3,4-DHPEA acetate	3,4-DHPEA-EDA	*p*-HPEA-EDA	3,4-DHPEA-EA	*p*-HPEA-EA	pinoresinol	1-acetoxypinoresinol	luteolin	apigenin	vanillic acid	*p*-coumaric acid	cinnamic acid	ferulic acid	Tyrosol derivatives	Lignans	Flavonoids	Phenolic acids	Total phenols	Orthodiphenols	Non-orthodiphenols
3,4-DHPEA	-																					
*p*-HPEA	^***^ 0.568	-																				
3,4-DHPEA acetate	–0.029	–0.150	-																			
3,4-DHPEA-EDA	0.085	0.013	0.055	-																		
*p*-HPEA-EDA	–0.046	0.153	0.044	^***^ 0.648	-																	
3,4-DHPEA-EA	^***^ 0.339	^***^ 0.294	^***^ –0.452	0.103	0.048	-																
*p*-HPEA-EA	* °.237	0.194	^**^ –0.267	0.103	0.096	^***^ 0.638	-															
pinoresinol	^***^ 0.374	^***^ 0.521	–0.108	0.033	0.151	^***^ 0.362	* °.21	-														
1-acetoxypinoresinol	^***^ 0.338	^***^ 0.397	–0.035	–0.138	–0.026	^***^ 0.37	0.157	^***^ 0.299	-													
luteolin	^**^ 0.238	^***^ 0.312	–0.068	* °.191	0.146	0.048	0.137	0.066	0.150	-												
apigenin	* °.177	* °.19	–0.076	0.145	0.163	0.023	* °.205	0.013	0.092	^***^ 0.828	-											
vanillic acid	^***^ 0.318	^***^ 0.414	0.012	–0.136	^**^ –0.232	–0.108	–0.044	0.105	^***^ 0.306	0.107	0.130	-										
*p*-coumaric acid	^***^ 0.306	^***^ 0.290	0.029	–0.046	–0.117	–0.002	0.077	0.018	* °.198	* °.202	0.117	^***^ 0.494	-									
cinnamic acid	^**^ 0.233	^***^ 0.426	^***^ –0.282	–0.001	* °.168	^***^ 0.760	^***^ 0.392	^***^ 0.533	^***^ 0.491	0.047	–0.040	–0.003	0.013	-								
ferulic acid	–0.002	0.073	–0.016	^***^ –0.287	^**^ –0.265	–0.113	–0.024	–0.142	* °.195	^***^ 0.350	* °.178	* °.275	^***^ 0.416	–0.065	-							
Tyrosol derivatives	^**^ 0.261	^**^ 0.251	^**^ –0.221	^***^ 0.722	^***^ 0.629	^***^ 0.720	^***^ 0.547	^***^ 0.302	* °.175	* °.171	0.144	* –°.197	–0.046	^***^ 0.545	^***^ –0.281	-						
Lignans	^***^ 0.407	^***^ 0.502	–0.063	–0.109	0.024	^***^ 0.431	* °.2	^***^ 0.565	^***^ 0.956	0.150	0.084	^***^ 0.297	* °.176	^***^ 0.588	0.125	^**^ 0.243	-					
Flavonoids	^**^ 0.228	^***^ 0.287	–0.073	* °.184	0.157	0.042	0.163	0.052	0.138	^***^ 0.985	^***^ 0.912	0.118	* °.183	0.023	^***^ 0.310	* °.169	0.136	-				
Phenolic acids	^***^ 0.373	^***^ 0.562	^**^ –0.233	–0.063	0.040	^***^ 0.626	^***^ 0.352	^***^ 0.486	^***^ 0.57	0.147	0.042	^***^ 0.404	^***^ 0.459	^***^ 0.867	* °.189	^***^ 0.396	^***^ 0.642	0.120	-			
Total phenols	^***^ 0.278	^**^ 0.273	^**^ –0.222	^***^ 0.711	^***^ 0.624	^***^ 0.728	^***^ 0.551	^***^ 0.321	* °.212	* °.194	0.163	* –°.179	–0.033	^***^ 0.562	^**^ –0.265	^***^ 0.999	^***^ 0.282	* °.192	^***^ 0.421	-		
Orthodiphenols	^***^ 0.318	^**^ 0.235	^**^ –0.264	^***^ 0.660	^***^ 0.413	^***^ 0.814	^***^ 0.544	^***^ 0.297	* °.211	0.162	0.112	–0.158	–0.021	^***^ 0.576	^**^ –0.247	^***^ 0.965	^**^ 0.273	0.153	^***^ 0.442	^***^ 0.965	-	
Non-orthodiphenols	0.088	^***^ 0.281	–0.046	^***^ 0.606	^***^ 0.944	^***^ 0.278	^***^ 0.389	* °.276	0.147	* °.211	* °.236	* –°.172	–0.051	^***^ 0.344	^**^ –0.225	^***^ 0.759	* °.211	^**^ 0.227	^**^ 0.23	^***^ 0.761	^***^ 0.565	-

Marked correlations are significant at: ^*^ *p*<0.05, ^**^ *p*<0.01, ^***^ *p*<0.001

Among the tyrosol and hydroxytyrosol derivatives, while low correlation coefficients were found for the simple phenols (*r* = 0.278 for 3,4-DHPEA and *r* = 0.273for *p*-HPEA) and total phenols, the four secoiridoid derivatives assessed were highly correlated to total phenols, showing those with a orthodiphenolic structure (3,4-DHPEA-EDA and 3,4-DHPEA-EA) the highest correlation coefficients (*r*>0.7). El Riachy et al. (2012a) found similar correlation coefficients for the secoiridoids with a monoaldehyde structure (3,4-DHPEA-EA and *p*-HPEA-EA) in the evaluation of segregating populations from crosses between different cultivars. However, they found non-significant correlations between total phenols content and the content of the secoiridoids with a dialdehyde structure (3,4-DHPEA-EDA and *p*-HPEA-EDA), despite the fact that 3,4-DHPEA-EDA was the main phenol in the oils. Total phenols also correlated significantly with cinnamic acid content (*r* = 0.562), which may point to the biochemical precursor of most of the phenols in the oils. As expected, tyrosol and hydroxytyrosol derivatives content correlated significantly to the four secoiridoid derivatives.

Significant correlation coefficients were also found among the individual phenols. In general, high correlation coefficients were found between compounds which include the 3,4-DHPEA moiety in their structures and those with the *p*-HPEA moiety. Thus, 3,4-DHPEA content was positively correlated to *p*-HPEA content (*r* = 0.568). Similarly, 3,4-DHPEA-EDA content correlated significantly to *p*-HPEA-EDA content (*r* = 0.648) and 3,4-DHPEA-EA content to *p*-HPEA-EA content (*r* = 0.638). However, this pattern of correlations was not always found by El Riachy et al. (2012a). On the other hand, the content of the secoiridoids with a dialdehyde structure (3,4-DHPEA-EDA and *p*-HPEA-EDA) displayed non-significant correlation coefficients to those with a monoaldehyde structure (3,4-DHPEA-EA and *p*-HPEA-EA). Interestingly, the content of 3,4-DHPEA acetate showed a significant negative correlation to the 3,4-DHPEA-EA content (*r* = −0.452) and to a lower extent to *p*-HPEA-EA (*r* = −0.267). These data may suggest a divergence in the metabolic pathway synthesizing 3,4-DHPEA acetate and 3,4-DHPEA-EA. Hypothetically, 3,4-DHPEA acetate would not be merely formed from an acetylation of 3,4-DHPEA but from a complex process involving cleavage, during the oil extraction process, of a unstable secoiridoid structure. This cleavage might occur during the decarboxylation of oleuropein aglycon, after deglucosylation, since our previous work showed no significant increases of 3,4-DHPEA acetate in *in vitro* deglucosylation of oleuropein or demethoxyoleuropein by pure olive β-glucosidase [13]. On the other hand, the content of the two flavonoids identified (apigenin and luteolin) was highly correlated (*r* = 0.828), as previously reported for other breeding progenies [25], [26].

**Figure 3:**
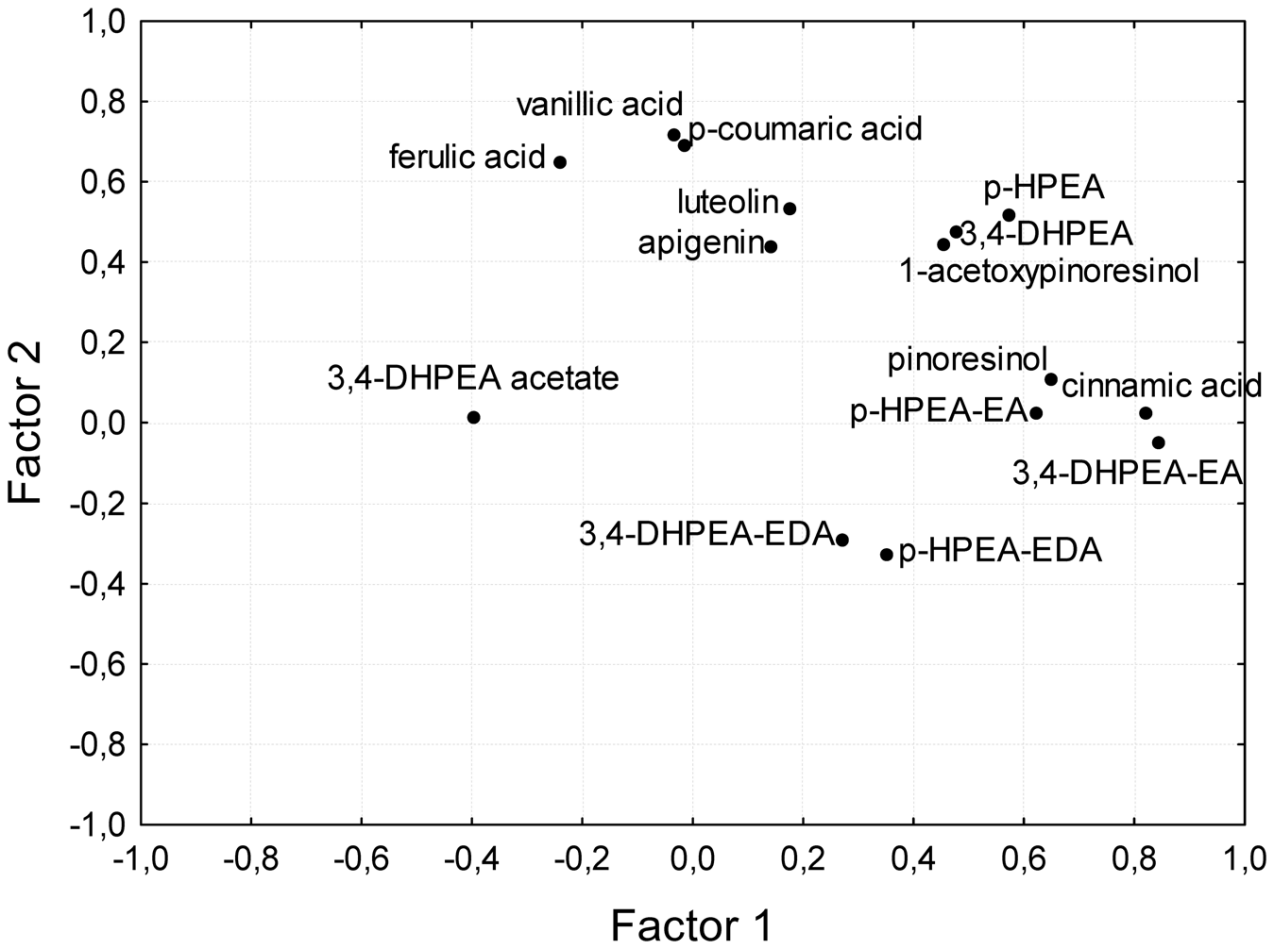
Factor analysis. Position of the main phenolic compounds in the oils from the Picual x Arbequina progeny on the first two factors using the normalized Varimax method.

Factor analysis was performed to explain the pattern of correlations within the different phenols assessed in the progeny oils. Table 2 displays the eigenvalues and the percentage of variance explained by those factors displaying eigenvalues higher than 1. As shown, they explained 64.59% of the variance. First factor explained 24.94% of the variance and second factor 16.46%. El Riachy et al. [25] found quite similar values (24% and 19% for factor 1 and 2, respectively) despite the fact that they assessed the contents of almost half of the phenols evaluated in this work. The coincidence in explaining the variance might be due to the fact that the main phenols, from a quantitative point of view, were evaluated in the oils in both works. Figure 3 shows the factor analysis bi-plots of the main VOO phenols considering the first two factors using the normalized Varimax method. As displayed, contents of pinoresinol, cinnamic acid, *p*-HPEA-EA, 3,4-DHPEA-EA and 3,4-DHPEA acetate were well explained by the first factor although for 3,4-DHPEA acetate was in opposite sense than the others. This is related to the negative correlation mentioned above between the content of this compound in the oils and the content of the secoiridoids with a monoaldehyde structure (3,4-DHPEA-EA and *p*-HPEA-EA) shown in Table 1. On the other hand the levels of vanillic and *p*-coumaric acids and the flavonoids lutein and apigenin were well explained by factor 2. Both *p*-HPEA and 3,4-DHPEA are explained fairly equally by the two factors, as seen in the first quadrant of the bi-plot, and the same occurs for *p*-HPEA-EDA and 3,4-DHPEA-EDA but in the fourth quadrant.

**Table 2:** Principal components analysis (eigenvalues) of the main phenolic compounds found in the progeny Picual x Arbequina oils.

Factor	Eigenvalue	Variance (%)	Cumulative (%)
1	3.74	24.94	24.94
2	2.47	16.46	41.40
3	2.02	13.46	54.86
4	1.46	9.73	64.59

**Figure 4:**
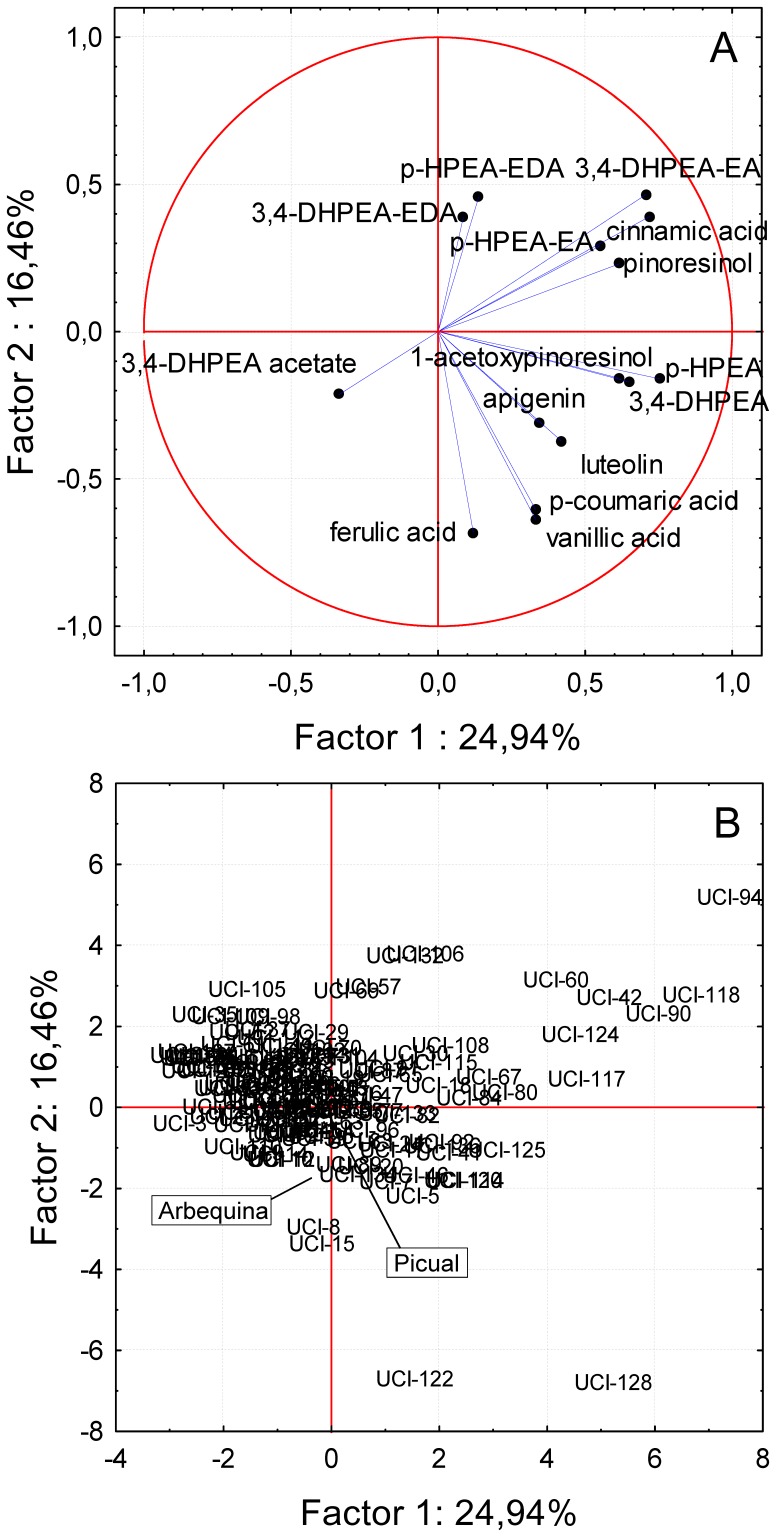
Principal component analysis of the main phenolic compounds. Bi-plot of the main phenolic compounds in the oils from the Picual x Arbequina progeny, including the genitors. Factors 1 and 2 explain 41.40 % of the data variation. A: vector distribution of the phenolic compounds, B: distribution of the genotypes from the progeny.

PCA was used to analyze the data for the phenols assessed in the Picual x Arbequina cross progeny oils (Figure 4). As mentioned above, the first two PCs carried a moderate amount of important information and accounted for 41.4% of the total variance. PCA bi-plots of the progeny oils showed strong associations between the secoiridoid compounds (Figure 4-A) and a number of progeny genotypes present in the first quadrant (Figure 4-B). Meanwhile, olive individuals closely associated to flavonoids, phenolic acids, simple phenols derived from tyrosol (*p*-HPEA and 3,4-DHPEA) are situated in the fourth quadrant, including the genitor Picual. Olive individuals associated to high levels of 3,4-DHPEA acetate are located mainly in the third quadrant.

**Figure 5:**
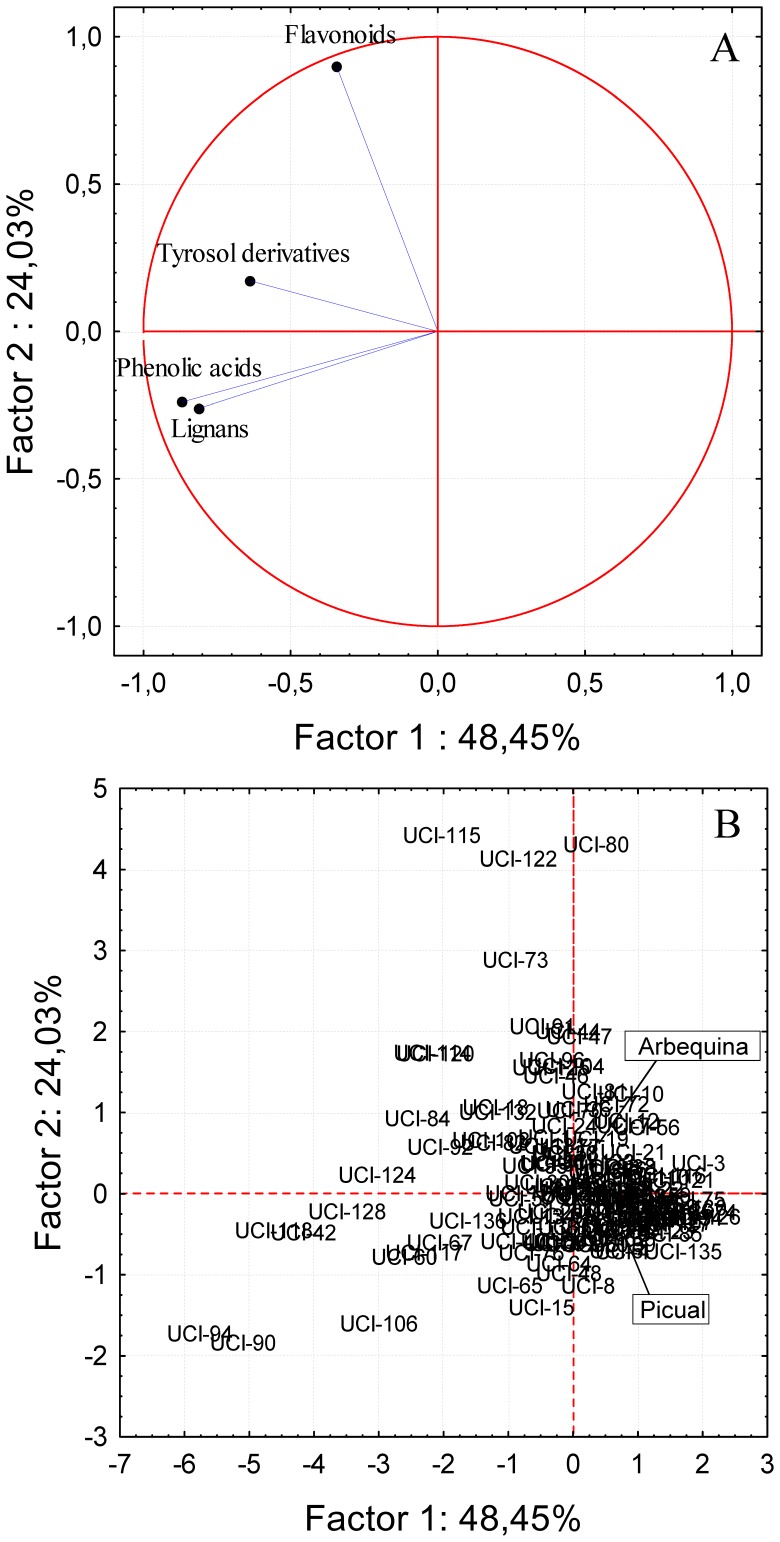
Principal component analysis of the main groups of phenolic compounds. Bi-plot of the main groups of phenolic compounds in the oils from the Picual x Arbequina progeny, including the genitors. Factors 1 and 2 explain 72.48 % of the data variation. A: vector distribution of the groups of phenolic compounds, B: distribution of the genotypes from the progeny.

PCA was performed considering as variables the four major groups of phenols in the progeny oils in order to distinguish genotypes especially rich in some of them (Figure 5). Genotypes having high content of lignans and phenolic acids are located in the third quadrant. According to the vector distribution plot in Figure 5-A, lignans and phenolic acids are closely related, so that when oils are rich in the phenols of one of these phenol groups, they have commonly high contents of phenols of the other group as well. Thus, it is possible to select genotypes from the progeny whose oils have a potential high phytoestrogenic activity (lignans) as well as a high level of antioxidants (phenolic acids) such as genotypes UCI-90, UCI-94, UCI-118 or UCI-42.

Genotypes having high content of flavonoids and tyrosol and hydroxytyrosol derivatives are situated in the second quadrant (Figure 5) and separated by Factor 2. Thus, the closer to the upper part of the first quadrant the more possible to found genotypes rich in flavonoids. This is the case of genotypes UCI-80, UCI-115 and UCI-122. As mentioned above, flavonoids are considered important health-promoting compounds because they are potent radical scavengers and antioxidants [40], [41].

**Figure 6:**
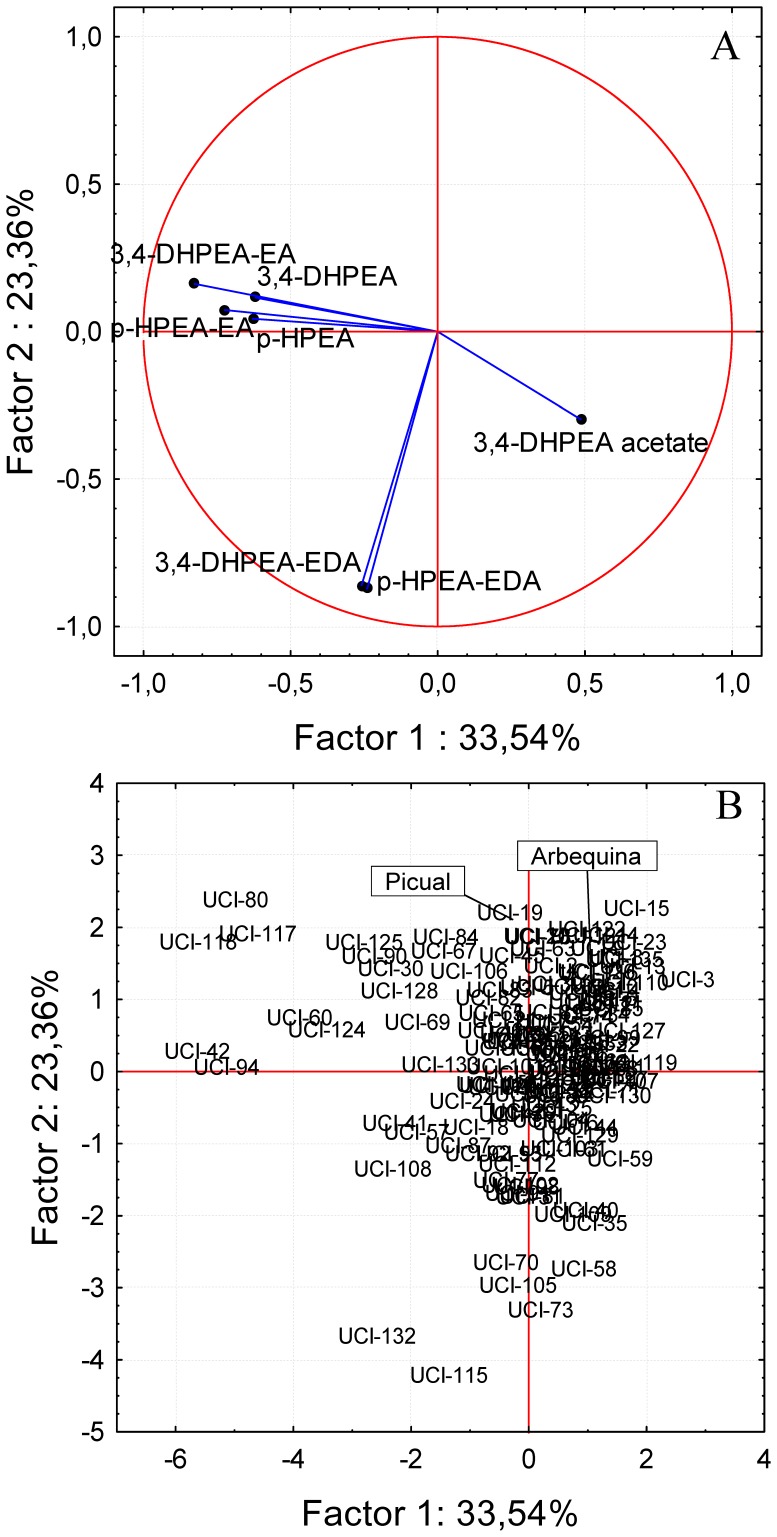
Principal component analysis of the tyrosol and hydroxytyrosol derived phenolic compounds. Bi-plot of the tyrosol and hydroxytyrosol derived phenolic compounds in the oils from the Picual x Arbequina progeny, including the genitors. Factors 1 and 2 explain 56.90 % of the data variation. A: vector distribution of the tyrosol and hydroxytyrosol derived phenolic compounds, B: distribution of the genotypes from the progeny.

Due to the nutritional and sensory implications of each of the tyrosol and hydroxytyrosol derivatives and their importance from a quantitative point of view, PCA was performed separately for this group of compounds (Figure 6). When considering as variables the content of the seven tyrosol and hydroxytyrosol derivatives evaluated in the progeny oils, the vectors of the secoiridoids with a monoaldehyde structure (3,4-DHPEA-EA and *p*-HPEA-EA) and those of simple phenols derived from tyrosol (*p*-HPEA-EDA and 3,4-DHPEA) are grouped together in the lower part of the second quadrant. This fact is related to the positive correlation coefficients found for those compounds as shown in Table 1. In the opposite location (fourth quadrant) is located the vector of 3,4-DHPEA acetate in good agreement with the negative correlation coefficients found (Table 1) for this compound when analyzed with respect to those secoiridoids and simple phenols mentioned above. Finally, the vectors of the secoiridoids with a dialdehyde structure (3,4-DHPEA-EDA and *p*-HPEA-EDA) are grouped together in the third quadrant, visibly separated from the rest of tyrosol and hydroxytyrosol derivatives.

This distribution of the vectors allows identifying in the third quadrant genotypes such as UCI-115, UCI-132, UCI-73, UCI-105or UCI-70, which presumably give rise to oils with remarkable health-promoting properties. These properties would be consequence of their high content of the antioxidant 3,4-DHPEA-EDA and the anti-inflammatory potential due to their elevated content of oleocanthal (*p*-HPEA-EDA) [1], [3]. However, the sensory aspects related to these oils should be also considered because of the importance from the point of view of the consumer acceptability. Despite most of these genotypes pointed out would give rise to oils with a mild bitter taste, considering the VOO bitterness calculation found by [6], they would be characterized by a high level of pungency due to the high level of *p*-HPEA-EDA. In this sense, Visioli and Bernardini [3] recommended consumers to be trained and informed on how to choose high-quality olive oils based on their organoleptic attributes. Oils rich in polyphenols are characterized by a bitter and pungent taste. The vector distribution displayed in Figure 6-A permits also to select genotypes whose oils would be characterized by a high health-promoting capacity, low pungency but highly bitter. These genotypes are those situated further left in the second quadrant in Figure 6-B such as genotypes UCI-42, UCI-94, UCI-118, UCI-117or UCI-80. Finally, in the fourth quadrant are located genotypes whose oils display a high content of 3,4-DHPEA acetate. This compound was proposed as a prodrug offering enhanced bioavailability for 3,4-DHPEA to the enterocytes for subsequent metabolism and basolateral efflux because it seems to be better absorbed than free 3,4-DHPEA [32]. Among the genotypes whose oils are rich in 3,4-DHPEA acetate were genotypes UCI-3, UCI-103, UCI-59, or UCI-5. Oils from these genotypes would be characterized by their low level of pungency and bitterness. Moreover, it would be possible to select genotypes whose oils would have a high level of 3,4-DHPEA acetate in combination with a high content of 3,4-DHPEA-EDA and oleocanthal (*p*-HPEA-EDA). These genotypes are situated in the lower part of the fourth quadrant, such as genotypes UCI-40, UCI-109, UCI-35, or UCI-58. These oils would have similar nutritional properties and better phenol availability than those in the third quadrant, but they theoretically would be perceived in the mouth less pungent and bitter.

The analysis of the data by PCA showed high variability in the seedling evaluated in any of the three years of harvest. Taking into account that the progeny and parents were grown in the same orchard, under the same edafo-climatic conditions, the oils extracted exactly in the same way, and that there was not any *a priori* criterion to select the genotypes being tested in each of the three sampling years, the fact that genotypes could not be grouped in terms of harvest year, considering any group of the variables selected for PCA shown in Figures 4, 5, 6 (Figure S1), might indicate that most of the variability found corresponds to genotype. However, El Riachy et al. [26] observed differentiation of groups when comparing a small amount of genotypes from the same cross in consecutive years. The cultivars Picual and Arbequina used as genitors are also represented in Figures 4, 5, 6. As shown, none of them are characterized for having a high content of a particular phenol or group of phenols despite their cross progeny displays a large degree of variability as seen also in Figure 1, widely transgressing their levels. This information on the correlations among phenolic compounds could be of interest for breeding programs aimed at producing new cultivars with high oil quality [8], [27].

The present study has demonstrated that it is possible to obtain a high degree of variability with a single cross of olive cultivars for the major phenolic compounds of VOO, which are main responsible for the sensorial and nutritional quality of this key element of the Mediterranean diet. This variability widely transgresses the genitor levels. In this sense, El Riachy et al. [25] suggested recently that it seems more effective to consider higher number of individuals within the same cross than using different crosses with small number of individuals. The use of multivariate analysis allowed to identify genotypes particularly interesting in terms of phenolic composition and deduced organoleptic and nutritional quality. Thus, the evaluation of phenolic compounds at seedling stage can be used in breeding programs to identify potential new olive cultivars, which give rise to oils with improved sensory and nutritional qualities.

## Supporting Information

Figure S1 **Principal component analysis of phenolic compounds according to the crop year.** Principal component analysis distribution of the genotypes from the Picual x Arbequina progeny taking as variables all the phenolic compounds (A), main groups of phenolic compounds (B), and the tyrosol and hydroxytyrosol derived phenolic compounds (C) in the oils. Symbols for the genotypes have different colors according to the crop year. Prediction ellipses are displayed for each crop year (coefficient = 0.95).
(TIFF)
